# Quantitative Proteomic Analysis Deciphers the Molecular Mechanism for Endosperm Nuclear Division in Early Rice Seed Development

**DOI:** 10.3390/plants12213715

**Published:** 2023-10-29

**Authors:** Jinmi Yoon, Cheol Woo Min, Jiyoung Kim, Gibeom Baek, Dohyeon Kim, Jeong Woo Jang, Ravi Gupta, Sun Tae Kim, Lae-Hyeon Cho

**Affiliations:** 1 Department of Biological Sciences, Inha University, Incheon 22212, Republic of Korea; jinmiyoon@inha.ac.kr; 2 Department of Biological Sciences and Bioengineering, Industry-Academia Interactive R&E Center for Bioprocess Innovation, Inha University, Incheon 22212, Republic of Korea; 3 Life and Industry Convergence Research Institute, Pusan National University, Miryang 50463, Republic of Korea; min0685@pusan.ac.kr; 4 Department of Plant Bioscience, College of Natural Resources and Life Science, Pusan National University, Miryang 50463, Republic of Korea; wldud8432@pusan.ac.kr (J.K.); bakki96@pusan.ac.kr (G.B.); ehgus1711@pusan.ac.kr (D.K.); com5222@pusan.ac.kr (J.W.J.); 5 College of General Education, Kookmin University, Seoul 02707, Republic of Korea; ravigupta07@ymail.com

**Keywords:** proteomic profiling, early seed development, CTP synthase, MCM proteins, protein stability, rice

## Abstract

Understanding the molecular mechanisms underlying early seed development is important in improving the grain yield and quality of crop plants. We performed a comparative label-free quantitative proteomic analysis of developing rice seeds for the WT and *osctps1-2* mutant, encoding a cytidine triphosphate synthase previously reported as the *endospermless 2* (*enl2*) mutant in rice, harvested at 0 and 1 d after pollination (DAP) to understand the molecular mechanism of early seed development. In total, 5231 proteins were identified, of which 902 changed in abundance between 0 and 1 DAP seeds. Proteins that preferentially accumulated at 1 DAP were involved in DNA replication and pyrimidine biosynthetic pathways. Notably, an increased abundance of OsCTPS1 was observed at 1 DAP; however, no such changes were observed at the transcriptional level. We further observed that the inhibition of phosphorylation increased the stability of this protein. Furthermore, in *osctps1-2*, minichromosome maintenance (MCM) proteins were significantly reduced compared with those in the WT at 1 DAP, and mutations in *OsMCM5* caused defects in seed development. These results highlight the molecular mechanisms underlying early seed development in rice at the post-transcriptional level.

## 1. Introduction

Seed development in angiosperms plays an important role in their life cycle and is tightly regulated at the molecular level [1]. The process of seed development in angiosperms is initiated by double fertilization, in which sperm cells fuse with a single egg cell and another sperm cell fuses with two polar nuclei, leading to the formation of a diploid embryo (zygote) and a triploid endosperm, respectively [1,2]. After double fertilization, the zygote produces a plant embryo for generating a whole new plant through elongation, cell division, and differentiation [3,4]. Endosperms normally undergo four developmental processes: syncytialization, cellularization, differentiation, and cell death [4]. The seeds of eudicots and monocots have different structures, particularly that of the endosperm. In eudicots, such as *Arabidopsis*, the embryo occupies the whole mature seed, and the endosperm is almost degenerated during seed maturation. In contrast, the endosperm constitutes the majority of the grain and is the seed’s primary storage organ in monocots such as wheat, maize, and rice [4,5,6].

Seed number and grain size are thought to be related to the evolution of various plant species [2,7]. It is important to understand the molecular mechanisms underlying early seed development to increase the grain yield and quality. Cereal endosperm is an important source of human nutrition and a major determinant of grain size and yield [8]. During syncytial endosperm development, the triploid endosperm nucleus undergoes rapid nuclear division without cytokinesis, leading to the formation of a multinucleate cell, the syncytium [6,8,9]. After nuclear division, the syncytial endosperm nuclei undergo cellularization via radial microtubule systems (RMSs) [10,11].

Rice is a major agricultural product that plays an important role in global food security by feeding more than half the world’s population. Rice endosperm is an important source of human nutrition and contains starch, protein, and various nutrients [12,13]. In rice, the seed develops within 1–3 days of fertilization, with early embryogenesis and rapid endosperm nuclear division. The regulatory mechanisms during early endosperm development have been extensively studied in *Arabidopsis* and involve epigenetic regulation and genomic imprinting, auxin signalling, microtubule-associated proteins, and transcription factors [14,15,16,17,18,19,20]. However, only a few genetic studies have been conducted on rice. The rice SNF2 helicase family protein ENL1, which is orthologous to human Plk1-Interacting Checkpoint Helicase (PICH), is responsible for extremely rapid nuclear division during syncytial endosperm development. Mutations in *ENL1* result in the failure of chromosome segregation and the development of strikingly enlarged syncytial endosperm nuclei [21]. Another protein, OsLFR, an ortholog of the *Arabidopsis* SWI/SNF chromatin-remodelling complex (CRC) component LFR, is essential for differentiation during early embryogenesis and endosperm nuclear division [22], while the OsMADS78 and OsMADS79 mediate cellularization of the endosperm nuclei. Both OsMADS78 and OsMADS79 interact with another type of I MADS-box protein, OsMADS89, which enhances their nuclear localization [23]. In addition, a recent study identified a long non-coding RNA, the MISSEN lncRNA gene, which negatively affects rice endosperm development and represents parent-of-origin expression. MISSEN lncRNA binds to a helicase family member, HeFP, which is highly expressed in *Arabidopsis* [24]. *OsCTPS1*, which encodes cytidine triphosphate synthase (CTPS), is required for early endosperm development during syncytial nuclear division by promoting nuclear spacing [13]. Although several factors involved in early seed development in rice have been identified, the molecular mechanisms underlying their functions have not been fully elucidated.

In recent years, RNA sequencing and other experimental approaches have been used to investigate gene expression during early seed and endosperm development [23] and to identify and study the roles of genes that affect seed size regulation [23,25,26]. However, there are limitations in studying the molecular mechanisms that occur during rapid nuclear division at the transcriptional level, and it is known that several factors affecting early seed development occur at the protein rather than the transcriptional regulation level. It is important to use proteomics analysis in addition to transcriptome analysis to investigate the molecular mechanisms occurring in seeds after double fertilization. Proteomic analysis provides a rich source for the study of detailed molecular biology, as it allows researchers to study the expression, protein modification, and interaction of proteins in a high-throughput manner [27,28]. In this study, we attempted to monitor the total protein profiles between the ovary just before fertilization and 1 d after pollination (1 DAP) of developing seeds to establish an initial data set to identify the mechanisms that regulate early seed development in rice. We also performed further analyses using *osctps1* mutants exhibiting an endosperm defect phenotype to elucidate the molecular mechanisms regulating endosperm development.

## 2. Results

### 2.1. OsCTPS1 Protein Accumulated in Developing Seeds at 1 DAP

**Figure 1:**
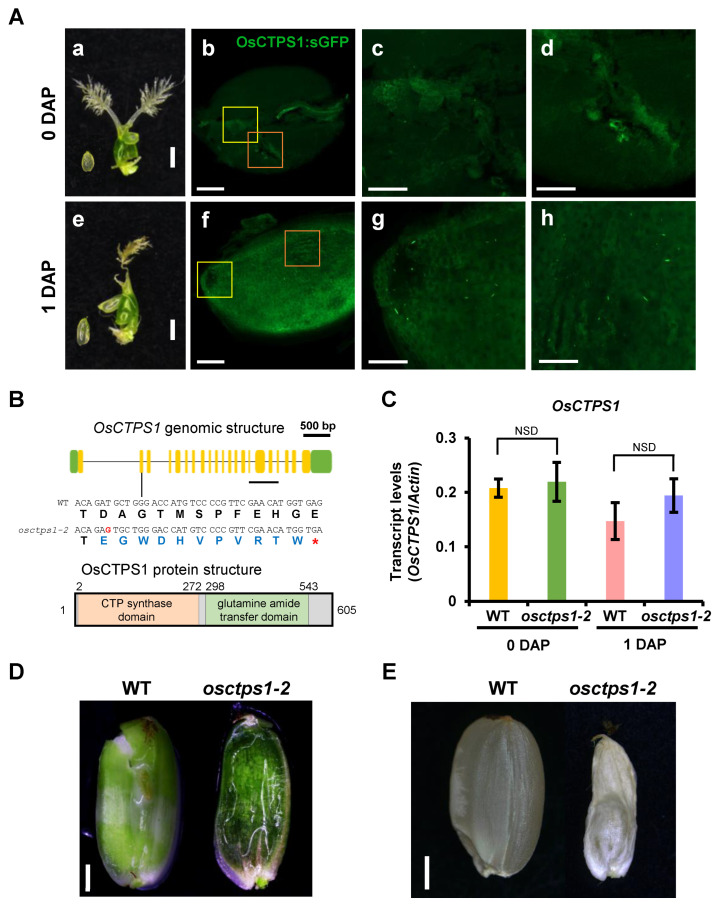
Protein accumulation of OsCTPS1 in the developing embryo sac at 1 day after pollination (DAP). (**A**) The OsCTPS1-sGFP fusion construct driven by the maize ubiquitin promoter was stably transformed. Embryo sacs from the transgenic plants were harvested at 0 DAP (**a**–**d**) and 1 DAP (**e**–**h**). c and d, enlargement images of yellow and orange boxed areas in panel b, respectively. g and h, enlargement images of yellow and orange boxed areas in panel f, respectively. Scale bars, 100 µm (**a**,**e**) and 50 µm (**b**–**d**,**f**–**h**). (**B**) Genomic structure of *OsCTPS1* and protein structure of OsCTPS1. The target site of the CRISPR/Cas9 system is also presented in the second exon region. The *osctps1-2* mutant showed premature termination, green boxs, UTR regions; yellow boxs, exon regions; *, premature termination codon. (**C**) Transcript levels of *OsCTPS1* in WT and *osctps1-2* mutants at 0 and 1 DAP. Each sample contains more than 10 ovaries or seeds; *n* = 4. NSD, no significant difference (*p >* 0.05). (**D**) Phenotype of WT and *osctps1-2* developing seeds at 10 DAP. Bar = 2 mm. (**E**) Phenotype of mature seeds of WT and *osctps1-2*. Bar = 2 mm.

We previously reported that the *endospermless 2* (*enl2*) rice mutant was defective in endosperm development [13]. The endospermless phenotype is caused by an alteration in rice *cytidine triphosphate synthase 1* (*OsCTPS1*), which encodes a rate-limiting step in the pyrimidine biosynthetic pathway that mediates the conversion of uridine triphosphate (UTP) to CTP. Despite its critical role in seed development, *OsCTPS1* was ubiquitously expressed in all examined tissues [13]. It has been suggested that the regulation of OsCTPS1 occurs at the post-translational level rather than at the transcriptional level. Using transgenic plants overexpressing OsCTPS1-sGFP, we examined the accumulation of the OsCTPS1-sGFP protein in developing seeds at 0 and 1 DAP (Figure 1Aa–h). Although the maize *ubiquitin* promoter leads to constitutively high expression in universal tissues regardless of tissue specificity, the OsCTPS1-sGFP fusion protein was detected at significant levels in developing seeds at 1 DAP but not at 0 DAP (Figure 1Af). At 1 DAP, GFP signals were observed in the embryo sac as macromolecular structures and bright spots, as previously reported [13]. These results suggested that OsCTPS1 proteins stably accumulate in the embryo sac during early endosperm development in developing seeds 1 DAP.

We have previously generated *osctps1* KO mutants using CRISPR/Cas9 [13]. However, the seed phenotypes of the mutants were severe and barely germinated. To investigate the molecular mechanism by which OsCTPS1 functions in seed development, we generated additional CRISPR/Cas9 KO mutants using the same target site [13]. We examined the mutations in the regenerated plants by sequencing the target site and selected one mutant for further experiments. The mutant *osctps1-2* displayed a 1 bp insertion in the second exon region of *OsCTPS1*. A single nucleotide insertion resulted in a premature termination codon in the gene (Figure 1B). *OsCTPS1* expression levels did not change significantly between 0 and 1 DAP in the WT and *osctps1-2* (Figure 1C), and we observed defects in endosperm development in *osctps1-2* as previously reported (Figure 1D,E) [13].

### 2.2. Label-Free Quantitative Proteomics Analysis in 0 and 1 DAP Rice Seeds

**Figure 2:**
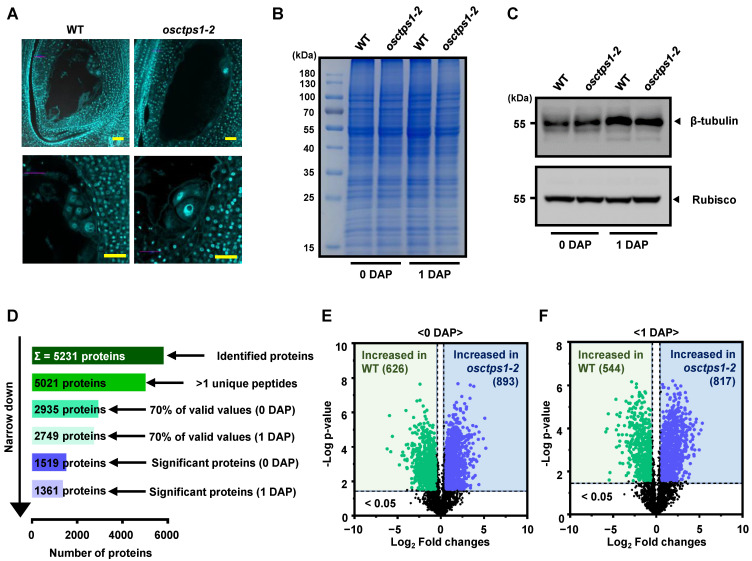
Comparison of differentially accumulated proteins between WT and *osctps1-2* in 0 and 1 DAP seeds. (**A**) Nuclear division phenotype in WT and *osctps1-2* at 1 DAP. Bars = 30 µm. (**B**) SDS-PAGE of total proteins isolated from 0 and 1 DAP seeds of WT and *osctps1-2* mutants. Loaded samples contained 30 µg of total proteins. (**C**) Western blot analysis using β-tubulin and Rubisco antibodies. Label-free quantitative proteomic analysis of 0 and 1 DAP seeds. (**D**) Schematic representation of the total number of proteins identified and the proteins validated by the narrow-down approaches. (**E**,**F**) A volcano plot of differently modulated proteins in WT and *osctps1-2* mutant seeds at 0 DAP (**E**) and 1 DAP (**F**).

We performed label-free quantitative proteomic analysis using WT and *osctps1-2* mutants from 0 (ovary just before pollination) and 1 DAP developing seeds to understand the developmental processes associated with early seed development in rice. At 1 DAP, the WT endosperm underwent rapid nuclear division with well-developed microtubule systems, whereas *osctps1-2* mutants showed aggregated endosperm nuclei with clumped microtubule structures (Figure 2A) [13]. After protein extraction, we examined the total protein in the SDS-PAGE gel using Coomassie blue staining and determined that the protein preparation for protein profiling was appropriate for subsequent analysis (Figure 2B). Since microtubule association is essential for nuclear division and migration during endosperm development, we examined the protein levels of β-tubulin, which is part of the microtubules, using western blot analysis and Rubisco protein as an internal control. The results showed that β-tubulin protein was slightly increased at 1 DAP in both WT and *osctps1-2* mutants compared to 0 DAP (Figure 2C).

**Figure 3:**
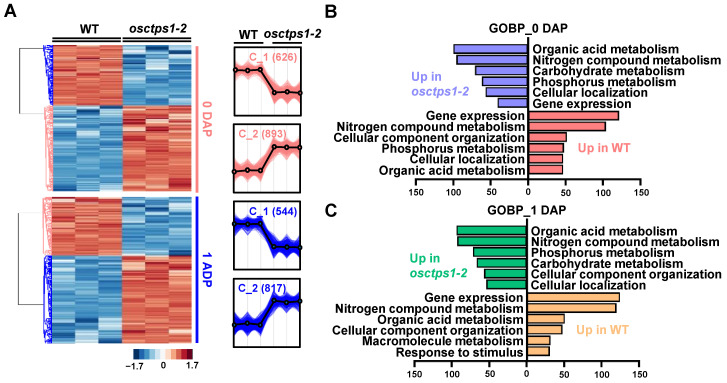
Comparison of differentially accumulated proteins between WT and *osctps1-2* in 0 and 1 DAP seeds. (**A**) Hierarchical clustering analysis of differentially modulated proteins identified using a label-free quantitative proteomic approach in 0 and 1 DAP samples. (**B**,**C**) Bar charts showing the representative gene ontology biological process (GOBP) term of significantly modulated proteins in WT and *osctps1-2* at 0 (**B**) and 1 DAP (**C**) using DAVID bioinformatics resources (GO enrichment).

In addition, we analysed the molecular changes occurring at 0 and 1 DAP in the developing seeds of the WT and *osctps1-2* mutants to understand the molecular mechanisms of initial rice seed development. A total of 5231 proteins were identified, of which 5021 showed more than one unique peptide and were considered significant hits (Figure 2D). Among these, 2935 and 2735 proteins showed 70% valid values in the three replicates of the 0 DAP and 1 DAP, respectively, and the subsequent application of Student’s *t*-test controlled using a Benjamini–Hochberg false discovery rate (FDR) threshold of 0.05 with ≥1.5-fold change showed 1519 and 1361 significantly modulated proteins in 0 DAP and 1 DAP seed samples, respectively (Figure 2D). Of these, 626 and 893 proteins accumulated in the WT and *osctps1-2* mutant rice seeds at 0 DAP, respectively (Figure 2E). In addition, of the 1361 significantly modulated proteins in 1 DAP sample set, 544 and 817 proteins were increased in the WT and *osctps1-2* mutants, respectively (Figure 2F). Moreover, hierarchical clustering analysis (HCA) revealed that significantly modulated proteins in both samples were segregated into two clusters (C_1 and C_2) (Figure 3A, Tables S2 and S3).

### 2.3. Functional Classification of Differentially Modulated Proteins in WT and osctps1-2 Seeds at 0 and 1 DAP Stages

Bioinformatics tools were used to characterise functional annotation using the DAVID bioinformatics resource to investigate the functions of all identified differentially modulated proteins (Figure 3B,C). Gene ontology (GO) enrichment analysis of increased abundance in 0 DAP of WT seed (C_1) showed that gene expression (GO:0010467, 122 proteins), including translation; organonitrogen compound biosynthesis (involved in nitrogen metabolism) (GO:1901566, 104 proteins), including peptide metabolism and biosynthetic process; cellular component biogenesis (GO:0044085, 52 proteins) involved in cellular component organization (GO:0071840); and phosphorus metabolism (GO:0006793, 48 proteins), including nucleotide metabolism, were the most enriched terms in the WT_0 DAP sample (Figure 3B, Table S4). By contrast, proteins exhibiting increased abundance in *osctps1-2* at the 0 DAP stage were mainly associated with organic acid metabolism (GO:006082, 100 proteins), including oxoacid and carboxylic acid metabolism; organonitrogen compound biosynthesis (GO:1901566, 96 proteins) involved in nitrogen compound metabolism; carbohydrate metabolism (GO:0005975, 71 proteins); and phosphorus metabolism (GO:0006793, 62 proteins), among others (Figure 3B, Table S4).

Moreover, similar GO enrichment annotations were observed in differentially modulated proteins of WT and *osctps1-2* seeds at the 0 and 1 DAP stages (Figure 3C, Table S5). In particular, gene expression (125 proteins), organonitrogen compound biosynthesis (involved in nitrogen compound metabolism) (120 proteins), organic acid metabolism (51 proteins), and cellular component organisation (48 proteins) were mainly annotated in the increased abundance proteins of WT at the 1 DAP stage (Figure 3C, Table S5). In contrast, proteins with increased abundance in *osctps1-2* at 1 DAP were related to organic acid metabolism (94 proteins), organonitrogen compound biosynthesis (93 proteins), phosphorus metabolism (72 proteins), carbohydrate metabolism (67 proteins), and cellular component biogenesis (57 proteins) (Figure 3C, Table S5). These results indicated that gene expression-associated proteins involved in protein metabolism (ribosomal activity), RNA processing and regulation, DNA synthesis and repair, and cell organization were specifically enriched in WT seeds at both the 0 and 1 DAP stages. Therefore, we compared the 0 and 1 DAP stages in WT seeds to understand the proteomic changes during development.

### 2.4. CTPS1 Function in Seed Development Associated with Protein Stability

**Figure 4:**
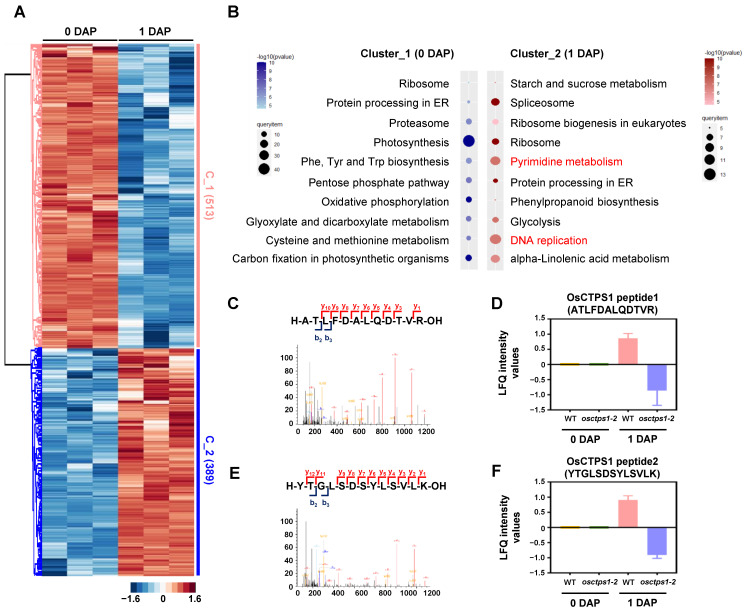
Quantitative proteomic analysis of 0 and 1 DAP of WT seeds. (**A**) Heat map images of significantly modulated proteins at 0 and 1 DAP in WT. (**B**) KEGG analysis of differentially accumulated proteins at 0 and 1 DAP seeds. Cluster1 included more accumulated proteins at 0 DAP seeds, and cluster2 included highly accumulated proteins in 1 DAP seeds. (**C**) The chromatographic peak of OsCTPS1 peptide 1 (ATLFDALQDTVR). (**D**) LFQ intensity of OsCTPS1 peptide 1 (ATLFDALQDTVR) at 0 and 1 DAP seeds in WT and *osctps1-2*; *n* = 3. (**E**) The chromatographic peak of OsCTPS1 peptide 2 (YTGLSDSYLSVLK). (**F**) LFQ intensity of OsCTPS1 peptide 2 (YTGLSDSYLSVLK) at 0 and 1 DAP seeds in WT and *osctps1-2*; *n* = 3.

To investigate the total proteome changes and abundance levels of OsCTPS1 during early development in WT seeds, we focused on the differentially abundant proteins between 0 and 1 DAP in WT seed samples (Figure 4A, Table S6). This analysis led to the identification of 902 significantly upregulated proteins. Among these, 513 proteins showed an increased abundance (C_1) at 0 DAP, whereas 389 proteins (C_2) showed an increased abundance at 1 DAP (Figure 4A, Table S6).

Kyoto Encyclopedia of Genes and Genomes (KEGG) pathway analysis of significantly modulated proteins showed that proteins at 0 DAP in WT seeds were mainly related to the photosynthetic pathway and may not be directly related to seed development (Figure 4B). Since cell division in the embryo and nuclear division in the endosperm occurred rapidly, we found a high accumulation of proteins involved in DNA replication and pyrimidine biosynthesis at 1 DAP (Figure 4B).

A more detailed analysis showed that the essential factor for endosperm development, OsCTPS1, was preferentially detected at 1 DAP rather than at 0 DAP (Figure 4C). Two peptides corresponding to OsCTPS1 were identified: ATLFDALQDTVR (peptide1) and YTGLSDSYLSVLK (peptide2) (Figure 4C). Both OsCTPS1 peptides were detected in the WT samples, but not in the *osctps1-2* mutants (Figure 4D). This may be related to the premature termination of OsCTPS1 in *osctps1-2* mutants, suggesting that an accumulation of the OsCTPS1 protein is responsible for early endosperm development.

### 2.5. OsCTPS1 Protein Accumulated in Developing Seeds at 1 DAP

**Figure 5:**
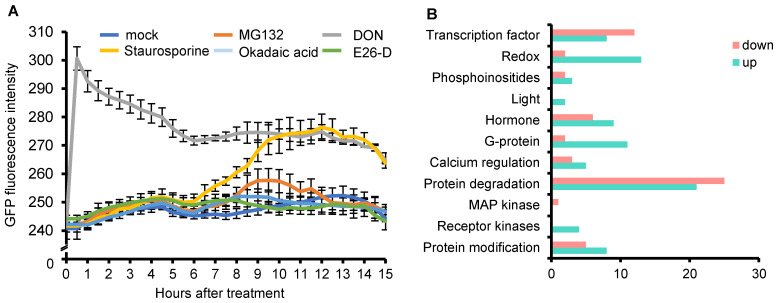
Regulation of OsCTPS1 protein stability. (**A**) Effect of treatment with mock or different signalling inhibitors on the stability of OsCTPS1-sGFP protein. Protoplasts were isolated from the OsCTPS1-sGFP OX transgenic plants and then incubated with 30 μM MG132, 2 μM staurosporine, 2 μM okadaic acid, 100 μM E64d, and 116.86 μM 6-diazo-5-oxo-L-norleucine (DON). After treatments, GFP fluorescence intensity was measured every 30 min for 15 h; *n* = 3. (**B**) MapMan overviews of regulation about differentially accumulated proteins in WT and *osctps1-2* in 1 DAP seeds.

Proteomic analysis revealed that OsCTPS1 accumulated stably in the embryo sac during early seed development. To investigate the regulatory mechanisms of OsCTPS1 protein stability, we isolated protoplasts from OsCTPS1-sGFP overexpressing transgenic plants in 10-day-old seedlings and examined the stability of the OsCTPS1-sGFP protein by measuring the intensity of GFP fluorescence during protoplast incubation. We treated the protoplasts with several signalling inhibitors, such as MG132, staurosporine, okadaic acid, and E64d, and the glutamine antagonist 6-diazo-5-oxo-L-norleucine (DON), which induces the formation of macromolecular structures of CTPS [29], as a control to analyse their effects on OsCTPS1 stability. MG132 is a potent, reversible, cell-permeable proteasome inhibitor. Staurosporine is a cell-permeable, potent, reversible, ATP-competitive inhibitor of protein kinases. Okadaic acid is a highly potent inhibitor of protein phosphatase 1 and 2A. In addition, E64d is an autophagy inhibitor that suppresses lysosomal proteases [30]. We observed that the fluorescence intensity increased rapidly after DON treatment (Figure 5A). DON treatment appeared to promote the macromolecular structure of CTP synthases and increased protein stability and GFP fluorescence intensity, which is consistent with previous reports [31]. In addition, an increase in OsCTPS1-sGFP protein stability was observed from approximately 6 h after incubation in cells treated with the protein kinase inhibitor staurosporine (Figure 5A), whereas no significant changes were observed for the other treatments. These results suggested that the inhibition of the phosphorylation of OsCTPS1 increases the stability of this protein. This pattern is similar to previous results, where it was reported that the kinase inhibitor staurosporine strongly stimulated filament formation by yeast CTP synthase [32].

We presumed that the accumulation of the OsCTPS1 protein was closely associated with early seed development and was affected by the regulation of phosphorylation. We compared the highly accumulated proteins at 1 DAP with those at 0 DAP in the WT seeds to investigate the factors affecting the accumulation of the OsCTPS1 protein. We then classified the functional groups from the proteomic analysis using protein modification and receptor kinases based on an overview of biological functions using MapMan analysis (Figure 5B). Future studies will focus on this protein as we expect that changes in its stability are likely due to changes in phosphorylation.

### 2.6. Minichromosome Maintenance (MCM) Proteins for DNA Replication Are Required for the Division of the Endosperm Nuclei

**Figure 6:**
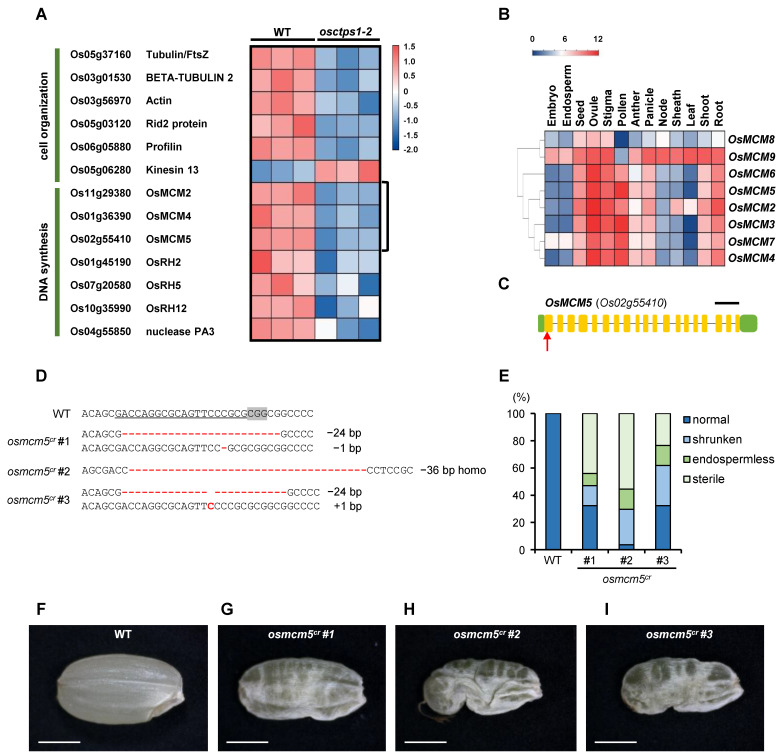
Comparison of differentially accumulated proteins between WT and *osctps1-2* in 1 DAP seeds and characterization of *OsMCM5* functions in seed development. (**A**) Heatmap images of protein expression involved in cell organization and DNA synthesis at 1 DAP in WT and *osctps1-2* mutants. Cell organization and DNA synthesis groups were selected for functions in endosperm nuclear division. (**B**) Expression patterns of rice MCM family genes in rice. (**C**) Schematic representation of the gene structure of *OsMCM5*. The red arrow indicates the target site of CRISPR/Cas9, green boxs, UTR regions; yellow boxs, exon regions. (**D**) Sequence alignment of the sgRNA target sequence with altered bases in three independent mutant lines. The target sequence is underlined, and the NGG PAM site is highlighted. Altered DNA sequences are indicated in red. (**E**) Different seed types caused by the mutation. More than 30 seeds were observed to check the seed phenotypes. The *osmcm5* mutant seeds were divided into four main types. (**F**–**I**) Phenotypes of WT (**F**) and *osmcm5* mutant seeds (**G**–**I**). Scale bars, 2 mm.

Because *osctps1* mutants exhibit defects in endosperm nuclear division, we examined the levels of proteins that were significantly reduced in *osctps1* mutants to understand the molecular mechanisms controlling endosperm nuclear division during the early stages of seed development. From the MapMan analysis, we identified two protein groups associated with cell organization and DNA synthesis, which could be related to endosperm nuclear division (Figure 6A). In the cell organization category, we found that microtubule association-related proteins, tubulin/FtsZ, β-tubulin, kinesin 13, MINI-CHROMOSOME MAINTENANCE (MCM) proteins, OsMCM2, OsMCM4, and OsMCM5 were significantly decreased in *osctps1-2* (Figure 6A). Among these, OsMCM5 had a slightly different protein structure from the other MCM proteins and lacked highly homologous proteins in the CAFRI-Rice database (Figure 6B) [33]. Therefore, to investigate the function of OsMCM5 during early seed development, we generated *osmcm5* null mutants using CRISPR/Cas9 (Figure 6C). The first exon was selected as the specific target site for *OsMCM5* mutations (Figure 6D). After genotyping by sequencing, three independent mutant lines were selected for further analysis (Figure 6E). All three mutant lines showed defects in seed development, including shrunken, endospermless, and sterile seeds (Figure 6F–I). These results suggested that OsMCM5 plays a critical role in early seed development, as its expression is increased at 1 DAP seeds compared to 0 DAP, as observed in our proteomics analysis. Finally, this is a good example of how genes encoding various proteins whose expression is altered during early seed development contribute to early seed development.

## 3. Discussion

Early seed development is a complex process involving cellular and molecular events such as double fertilization, embryogenesis, and endosperm development. The syncytial endosperm stage, which is the early stage of endosperm nuclear division, is characterised by a rapid increase in the number of nuclei, with the developing embryo sac giving rise to as many as 8000 nuclei from a single endosperm nucleus within 1–2 DAP, representing a dramatic and rapid developmental change [13,34]. A detailed understanding of the early stages of seed development, both in the embryo and the endosperm, may enable us to develop ways to increase rice grain yield and quality efficiently. After double fertilization, multiple proteins related to spliceosome, ribosome, pyrimidine metabolism, and DNA replication were significantly accumulated. Although previous studies have shown that DNA helicases are important for early seed development, the detailed mechanisms underlying these processes are poorly understood.

In the total proteomic analysis of the 0 and 1 DAP seeds, we focused on OsCTPS1, a protein associated with endosperm development. We have previously reported that *OsCTPS1* is essential in early seed development [13]. Both previous research and the total proteomic data reported in this study indicate that the accumulation of OsCTPS1 protein is particularly high during early seed development in rice, suggesting the presence of factors that control the stability of OsCTPS1 proteins. Regulatory mechanisms that affect CTP synthase enzyme activity and protein stability have been reported. The common regulatory mechanism between CTP synthase enzyme activity and filament formation has been previously investigated, and the results showed that both the enzyme activity and filament formation of CTP synthase are regulated by UTP [35]. Similar concentrations of UTP are required for increased enzyme activity and enhanced filament formation, suggesting a mutual dependence between the two processes. The results of this study also showed that enzyme activity increased at low concentrations of CTP synthase protein but decreased at higher concentrations, likely due to filament formation at high concentrations of the protein. Finally, a previous study has reported that certain structural domains within the CTP synthase protein play important roles in filament formation and regulation. Understanding the functions of these domains may provide new insights into the regulation of CTP synthase activity and filament formation [35]. In addition, cryo-EM and structural studies have identified key residues of CTP synthase and domains that bind to substrates, products, and allosteric regulators [24]. Different ligands bind to distinct regions of the enzyme, with some residues playing important roles in determining the specificity and affinity of ligand binding [24]. In addition, the binding of certain ligands can lead to conformational changes in CTP synthase. For example, the binding of ATP and UTP to specific domains of CTP synthase promotes filament formation and inhibits enzyme activity [24]. CTP synthase activity is also regulated by its phosphorylation status, with phosphorylation at a specific position increased by protein kinases A and C [36,37,38]. While CTP synthases are phosphorylated by protein kinase A and protein kinase C in yeast, the phosphorylation is mediated by glycogen synthase kinase 3 (GSK3) in human cells, and phosphorylation actually inhibits the function of CTP synthase [38]. In addition, the filamentous macromolecular structure of CTP synthase is strongly induced by the kinase inhibitor, staurosporine [32]. Based on these results, it is expected that filamentous macromolecular structure formation, enzyme activity, and protein stability of OsCTPS1 in rice are affected by phosphorylation. However, further research is required to investigate these effects in detail.

Based on total proteome profiling, we aimed to elucidate the molecular mechanisms underlying nuclear division during endosperm development. Therefore, we were interested in minichromosome maintenance (MCM) proteins and DEAD-box RNA helicase (RH) homologous proteins, which showed significantly decreased protein expression in *osctps1-2* mutant compared to the WT at 1 DAP, which is defective in nuclear division during early endosperm development. The MCM complex may function as a DNA helicase, opening a replication fork to maintain DNA replication [39,40]. In a previous report, a mutation in *AtMCM2* was shown to be lethal at an early stage of embryogenesis, and it was suggested that AtMCM2 affects cell proliferation in the root meristem through a gain-of-function approach. We found that *osmcm5* mutants had defects in seed development. This provides a compelling illustration of how genes encoding diverse sets of proteins whose expression changes during the early stages of seed development are actively involved in early seed development. We further attempted to find a link between CTP synthase and MCM proteins based on previously published studies; however, this was unsuccessful. Since endosperm nuclear division is defective in *osctps1* mutants, it is possible that MCM proteins involved in DNA replication are reduced, and we plan to investigate this further. Although several questions remain unanswered, we anticipate that functional analysis will be more important at the level of protein regulation than at the transcriptional level.

## 4. Materials and Methods

### 4.1. Plant Materials and Growth Conditions

Japonica rice (*Oryza sativa* cv. Nipponbare) plants were grown in an experimental paddy field at Pusan National University, Republic of Korea (35°45′ N). Seeds were germinated on 1/2 Murashige and Skoog’s medium containing 3% sucrose or soil, as previously reported [41].

### 4.2. Vector Construction and Rice Transformation

To generate knockout mutants using the CRISPR/Cas9 method, we examined specific target sites in the genome sequences of *OsCTPS1* and *OsMCM5* using the CRISPRdirect web tool (http://crispr.dbcls.jp, accessed on 5 January 2022) [42] to search for effective protospacer adjacent motifs (PAMs) and avoid off-target sites. The target sequences of *OsCTPS1* (F: 5′-GGCACCTTACCTTAACACAGATGC-3′; R: 5′-AAACGCATCTGTGTTAAGGTAAGG-3′) and *OsMCM5* (F: 5′-GGCAGACCAGGCGCAGTTCCCGCG-3′; R: 5′-AAACCGCGGGAACTGCGCCTGGTC-3′) were inserted into the CRISPR/Cas9 binary vector pRGEB32 to express the guide RNA and Cas9 enzyme.

### 4.3. RNA Isolation and Quantitative RT-PCR Analyses

Total RNA was isolated from rice leaf blades using RNAiso reagent (Takara, Shiga, Japan). The first cDNA was synthesised from 2 μg of total RNA, using Moloney murine leukaemia virus reverse transcriptase (Promega, Madison, WI, USA; http://www.promega.com, accessed on 10 May 2023) with 10 ng of the oligo (dT)_18_ primer and 2.5 mM deoxyribonucleotide triphosphate. Quantitative RT-PCR was performed using a Rotor-Gene Q instrument system (QIAGEN, Valencia, CA, USA), and the synthesised cDNAs were amplified using SYBR Premix Ex Taq (Takara) as previously reported [43]. Rice *Ubi1* was used as an internal control. All experiments were performed at least thrice with four or more samples at each time point. All primers used for qPCR are listed in Supplementary Table S1.

### 4.4. Quantification of GFP Fluorescence Intensity

Protoplasts were isolated as previously described [44] with minor modifications. Briefly, etiolated leaves of 10-day-old *OsCTPS1-sGFP* overexpressing transgenic plants were collected and cut into ~1 mm slices using a sharp blade. The sliced samples were then incubated in a solution (1.5% *w*/*v* cellulase RS, 0.5% *w*/*v* macerozyme, 0.1% *w*/*v* pectolyase, 0.6 M mannitol, 5 mM MES pH 5.7, 10 mM CaCl_2_, and 0.1% *w*/*v* BSA) for 4 h with gentle shaking in the dark. After incubation, an equal volume of a solution (117 mM KCl, 82 mM MgCl_2_, and 85 mM CaCl_2_) was added, the cells were harvested, and their protoplasts were resuspended in a solution (0.4 M mannitol, 70 mM KCl, 5 mM MgCl_2_, and 0.1% *w*/*v* MES pH 5.7) at a density of 3 × 10^6^ cells mL^−1^, as quantified using a haemocytometer. The protoplasts were then incubated with 0.1 μM MG132, 10 nM staurosporine, 100 nM okadaic acid, 10 μg/mL E64d, and 4 μg/mL 6-diazo-5-oxo-L-norleucine (DON) individually. During treatment, GFP fluorescence intensity was measured every 30 min over a 15 h period using a Glomax-Multi Detection System (Promega, Madison, WI, USA; http://www.promega.com, accessed on 10 May 2023).

### 4.5. Total Protein Isolation and In-Solution Trypsin Digestion

To isolate total protein from developing rice seeds, trichloroacetic acid (TCA)/acetone precipitation was performed as previously described [45]. Briefly, 0.1 g of each finely ground powder was homogenised with 10 mL of ice-cold RIPA extraction buffer [150 mM sodium chloride (NaCl), 50 mM Tris-HCl, pH 8.0, 1% Nonidet P-40 (NP-40), 0.5% sodium deoxycholate (SDC), 0.1% sodium dodecyl sulfate (SDS)] and centrifuged at 15,922× *g* for 10 min at 4 °C. The collected clear homogenate was then precipitated with 4 volumes of 12.5% TCA/acetone containing 0.07% (*v*/*v*) *β*-mercaptoethanol overnight at −20 °C. Finally, the resulting pellets were further washed with 80% acetone containing 0.07% *β*-mercaptoethanol with centrifugation at 15,922× *g* for 5 min at 4 °C and stored at −20 °C for further analysis.

Protein digestion was performed using filter-assisted sample preparation (FASP) [46,47]. Briefly, acetone-precipitated proteins (300 μg) were dissolved in 30 μL of denaturation buffer [4% SDS and 100 mM 1,4-dithiothreitol (DTT) in 0.1 M tetraethylammonium tetrahydroborate (TEAB), pH 8.5]. Proteins were denatured after sonication for 3 min and heating at 99 °C for 30 min and loaded onto a 30K spin filter (Merck Millipore, Darmstadt, Germany) after dilution to a final volume of 300 μL in urea buffer [8 M urea in 100 mM TEAB, pH 8.5]. The buffer was then replaced with 300 μL of urea buffer by centrifugation at 14,000× *g* for 10 min at 20 °C to remove the SDS; this step was performed three times. In addition, cysteine alkylation was performed by adding 200 μL of alkylation buffer [50 mM iodoacetamide (IAA), 8 M urea in 0.1 M Tris-HCl, pH 8.5] for 1 h at room temperature in the dark and exchanging the buffer with urea buffer to TEAB [50 mM TEAB, pH 8.5] in a spin filter unit. Trypsin (enzyme-to-substrate ratio [*w*/*w*] of 1:100) dissolved in 5% acetonitrile (ACN) was then added for digestion and incubated at 37 °C overnight. Digested peptides were collected using centrifugation, and concentrations were measured using the Pierce Quantitative Fluorometric Peptide Assay (Thermo Fisher Scientific, Waltham, MA, USA) according to the manufacturer’s instructions.

### 4.6. Peptide Desalting and High pH Reversed-Phase Peptide Fractionation

The digested peptide samples were desalted using an HLB OASIS column and lyophilised using vacuum centrifugation, as described in previous reports and according to the manufacturer’s instructions [48]. After desalting, the dried peptides were re-dissolved in 200 μL of loading buffer [15 mM ammonium formate, 2% ACN] and loaded onto a proprietary STaGE tip prepared by packing C18 Empore disk membranes (3M, Bracknell, UK) at the bottom and POROS 20 R2 reverse-phase resin in the 200 μL yellow tip. Before loading the peptide samples, the STaGE tip was washed with 100% methanol and 100% ACN and equilibrated with the loading buffer. After peptide loading, three fractions of each sample were eluted with a pH 10 buffer solution containing 5, 10, 15, 20, 25, 30, 35, 40, 60, 80, or 100% ACN [49]. Finally, each fraction was lyophilised using vacuum centrifugation and stored at −80 °C for further LC-MS/MS analysis.

### 4.7. LC-MS/MS Analysis

Briefly, the peptide fractions obtained were analysed with a liquid chromatography-tandem mass spectrometry (LC-MS/MS) system combined with a UHPLC Dionex UltiMate 3000 (Thermo Fisher Scientific, Waltham, MA, USA) instrument and a QExactive Orbitrap High-Resolution Mass Spectrometer (Thermo Fisher Scientific, Waltham, MA, USA) as previously described [50]. The fractionated peptides were dissolved in solvent A [2% ACN and 0.1% formic acid] and separated by reverse-phase chromatography using a Dionex UltiMate 3000 UHPLC instrument (Thermo Fisher Scientific). A two-column system with a trap column (Thermo Fisher Scientific, Acclaim PepMap 100 trap column, 100 μm × 2 cm, nanoViper C18, 5 μm, 100 Å) and an analytical column (Thermo Fisher Scientific, Acclaim PepMap 100 capillary column, 75 μm × 15 cm, nanoViper C18, 3 μm, 100 Å) was used for peptide separation. Peptide samples were separated using a 90 min nonlinear gradient from 2% to 35% solvent B [100% ACN and 0.1% formic acid]. The electrospray ionization source was coupled to a QExactive MS instrument (Thermo Fisher Scientific, Waltham, MA, USA). The resulting peptides were electrosprayed using a coated silica-emitting tip (Scientific Instrument Service, Amwell Township, NJ, USA) at an ion spray voltage of 2000 eV. Mass spectra were measured in data-dependent mode for the 15 most abundant peaks (Top15 method). The precursor ions were acquired at a resolution of 70,000 at 200 *m*/*z* in the mass range of 350–1800 *m*/*z*. The automatic gain control (AGC) was set to 3 × 10^6^, and the MS/MS isolation window was 1.2 *m*/*z*. Ion activation/dissociation with higher-energy C-trap dissociation (HCD) scans was acquired at 35,000 resolution and 32 normalised collision energies (NCE). The AGC target for MS/MS analysis was 2 × 10^5^. The maximum ion injection times for the survey and MS/MS scans were 30 and 120 ms, respectively. Subsequently, the proteomic data were deposited in the ProteomeXchange Consortium via the PRIDE partner repository with the dataset identifier PXD043162 [51].

### 4.8. Data Analysis

MaxQuant software (version 2.0.3.0) was used to analyse the acquired LC-MS/MS data as described previously [49,52]. The three replicates of the MS/MS spectra were matched against the *Oryza sativa* database (MSU v6.0, 67,393 entries). Label-free quantification (LFQ) data were processed using an Andromeda’s default precursor mass tolerance of 20 ppm for the first search and 4.5 ppm for subsequent searches. LFQ data were searched based on 0.5 Da of a product mass tolerance with a maximum of two missed tryptic digests. Carbamidomethylation of cysteine residues, acetylation of lysine residues, and oxidation of methionine residues were selected as fixed and variable modifications. The FDR, which was set at 1% for peptide identification, was determined using a reverse nonsense version of the original database. Sequential statistical analyses of the LFQ data were performed using Perseus (ver. 1.6.15.0) as previously described [53]. Missing value imputation of protein intensities was performed by calculating normal distribution (width: 0.3, downshift: 1.8), HCA, and a multiple-sample test (one-way ANOVA) controlled by Benjamini–Hochberg FDR threshold of 0.05, and the determination of significant differences in the protein abundance (≥1.5-fold change) between different samples was calculated using Perseus software. Functional classification, including GO enrichment, subcellular localization, and MapMan analysis, was performed using the CELLO2GO (http://cello.life.nctu.edu.tw/cello2go/; accessed on 26 October 2023) and MapMan (https://mapman.gabipd.org/mapman; accessed on 26 October 2023) software, respectively [54].

## Data Availability

Mass spectrometry proteomics data were deposited in the ProteomeXchange Consortium via the PRIDE partner repository with the dataset identifier PXD043162 (Reviewer account details: Username: reviewer_pxd043162@ebi.ac.uk; Password: 1O8DLMJZ) [50].

## Supplementary Materials

The following supporting information can be downloaded at: https://www.mdpi.com/article/10.3390/plants12213715/s1, Table S1: List of primers used in this study; Table S2: List of differentially expressed proteins in WT and *osctps1*-2 in 0 DAP seeds; Table S3: List of differentially modulated proteins in WT and *osctps1*-2 in 1 DAP seeds; Table S4: GO enrichment analysis of identified proteins in 0 DAP seeds; Table S5: GO enrichment analysis of identified proteins in 1 DAP seeds; Table S6: List of differentially expressed proteins in WT seeds at 0 and 1 DAP.
